# Supplementary material for: Patterns of Intron Gain and Loss in Fungi
Source: PLoS Biol. 2004 Nov 30;2(12):e422. doi: 10.1371/journal.pbio.0020422 (PMC532390; doi:10.1371/journal.pbio.0020422)
Supplement: Table S1 — Also available at http://genes.mit.edu/NielsenEtAl/. (4.3 MB ZIP). [file pbio.0020422.st001.zip › NielsenEtAl/html/1387.html]

AN4395.1.NCU08939.1.MG06191.1.FG10091.1


```
 CLUSTAL W (1.82) Multiple Sequence Alignments - Introns Inserted


Sequence 1: NCU08939.1	404 aa
Sequence 2: MG06191.1	404 aa
Sequence 3: AN4395.1	342 aa
Sequence 4: FG10091.1	437 aa
Alignment Length: 443 aa
Number Identitical Residues: 211 aa
Alignment Score (without introns) 10965


MG06191.1 	-------~----------------~----------MLLPKGGLNWKAARGSLPPSRAIWT
NCU08939.1	-------~----------------~----------MLLPKGGVSWKAARAQLPPARALWA
FG10091.1 	MDNVINR2PLAQTPTTLKSPAITP0WLGRLLDEFAMLSSNGRISWKAAKAHMPQTRALRS
AN4395.1  	-------~----------------~-----------------------------------
          	                                                            

MG06191.1 	FVTRTRFLLLVALSGVFLLLWRGIRSSASEMQS2FYCWGPSKPPMDMTVNEQAAWNAHMQ
NCU08939.1	ILTKTRFLLLVAVTGVVLLLWRGISTGASEMQS2FYCWGPSKPPMEMTLNEQQAWNAHLH
FG10091.1 	NLTRSRLLLILGLTGIVMLLYRGLRGAPPGPQN2VRCWGPNKSPMEMTPNEHALWNAHMQ
AN4395.1  	---------------------------------~----------MDMSLNEITEWTAHAQ
          	                                            *:*: **   *.** :

MG06191.1 	TPVIFNHHMPLEINSSTIIHHDLNPIKSTPKAVANEERVLILTPLKDAAPYLSKYFELIA
NCU08939.1	TPVIFNHHAPLEINSSTISHVDLNPVKSTTKAVANEERILILTPLKDASRYLSKYFELIA
FG10091.1 	TPVLFNHHKPLKIDSSSIQHIDLNPITSSPQAVVRNERILILTPLKDAAPFLKNYFKLIA
AN4395.1  	TPVLFNYHEPYEVNSSSIKNVDLNPIKSTNQAVQNRERVLILTPLRDASFHLNRYFDLIY
          	***:**:* * :::**:* : ****:.*: :** ..**:******:**: .*..**.** 

MG06191.1 	ELTYPHHLIDLAFLVGDSTDDTLAVLASEIDRIQKRSDNVPFRSATIVEKDFNFKLSQDV
NCU08939.1	ELTYPHHLIDLAFLVSDSTDDTLAVLASELDRIQKRPDQIPFHSAMVVEKDFDFKLSQNV
FG10091.1 	ELTYPHRLIDLAFLVSDSKDDTLAVLASELDHLQKRKDEVPFNSITIVEKNFHFHLSQDA
AN4395.1  	KLTYPHELIDLAFLVGDTKDDTLATLASELQRIQDQGDKVAFRSATIIQKDFGTDFEMNV
          	:*****.********.*:.*****.****::::*.: *::.*.*  :::*:*  .:. :.

MG06191.1 	KDRHGFEAQAPRRKNMGRARNYLLYTALKPEHSWVYWRDVDIVDSPKAVLEDFVAHDKDI
NCU08939.1	EERHSFAAQGPRRKAMGRARNYLLSAALKPEHSWVYWRDVDIVDSPKKILEDFIAHDKDI
FG10091.1 	KQRHGFEVQAPRRKAMAKARNYLLATALKPEHSWVYWRDVDIEEAPRRILEDFIAHDRDI
AN4395.1  	EDRHSFAAQGPRRKAIGRARNYLLYSALKPDHSWVYWRDVDIVDSPDTILEDFIAHDKDI
          	::**.* .*.**** :.:****** :****:*********** ::*  :****:***:**

MG06191.1 	LVPN~IWFHRYKDGKDIEGRF1DYNSWIESDKGRKLAASLDKDVVLAEG1YKQYDTGRTY
NCU08939.1	LVPN1IWFHRYQNGRDIEGRF1DYNSWVESDKGRRLASSLDKDVVLAEG1YKEYDTGRTY
FG10091.1 	LVPN1IWFHRFEKGVDIEGRF1DYNSWIESDKGRKLANSLPKDTIIVEG~YKEFDTGREY
AN4395.1  	LVPN1IWFHRYKDGVDIEGRF1DYNSWIESDKGRRLRATLDPDTILAEG~YKEYDTGREY
          	**** *****::.* ****** *****:******:*  :*  *.::.** **::**** *

MG06191.1 	MARMGDWREDPTKEIELDGIGGVNIVVKADVHRSG1INFPCYAFENQAETEGFAKMARRA
NCU08939.1	MARMGDWRDDKDVEIELDGIGGVNILVKADVHRSG1INFPCYAFENQAETEGFAKMAKRA
FG10091.1 	MCRMGDWRENKDVELKLDGIGGVNILVKADVHRSG1INFPAYAFENQAETEGFAKMAKRA
AN4395.1  	LVGMGDWRNDKDKEVELDGIGGVNIVVKADVHRSG1INFPAYAFENQAETEGFARMAKRA
          	:  *****::   *::*********:********* ****.*************:**:**

MG06191.1 	GYQVIGLPNYVVWHIDTDEKAGNV------
NCU08939.1	GYQVIGLPNYVVWHIDTEEKGGNA------
FG10091.1 	GYQVIGLPNYVVWHYDTQEKGGNL------
AN4395.1  	GYQVIGLPNYVVWHIDTDEKPGNLGDRKAY
          	************** **:** ** .. .:
```
